# Combined IFN-β and PLT Detection Can Identify Kawasaki Disease Efficiently

**DOI:** 10.3389/fped.2021.624818

**Published:** 2021-04-22

**Authors:** Kan Huijuan, Dong Yaping, Wang Bo, Hou Miao, Qian Guanghui, Yan Wenhua

**Affiliations:** ^1^Department of Cardiology, Children's Hospital Soochow University, Suzhou, China; ^2^Department of Pediatric, The Affiliated Suzhou Hospital of Nanjing Medical University, Suzhou Municipal Hospital, Suzhou, China

**Keywords:** Kawasaki disease, IFN-β, PLT, biomarker, STING pathway

## Abstract

**Objective:** To evaluate the value of combined interferon β (IFN-β) and platelet (PLT) detection for Kawasaki disease (KD) identification.

**Methods:** Forty-four children who were newly diagnosed with KD were selected as the KD group. They were divided into acute phase of KD and subacute phase of KD. They were also separated into groups with and without coronary artery disease (CAD) (CAD+ and CAD–, respectively). Meanwhile, 44 children hospitalized with febrile disease and 44 healthy children were selected as a febrile control group and normal control group, whom were attended to at Children's Hospital of Soochow University at the same time. We detected the concentration of IFN-β and PLT of peripheral blood serum for all three groups and analyzed the difference.

**Results:** At acute and subacute phases of KD, both IFN-β and PLT are higher than both the febrile control group and healthy control group, especially at subacute phase; the difference between groups was statistically significant, *P* < 0.05. Receiver operating characteristic (ROC) curve showed that the areas under the ROC curve (AUCs) of IFN-β and PLT at acute phase of KD were 0.81 and 0.72, respectively; the sensitivity and specificity were 97.22 and 63.64%, and 57.89 and 73.86%, respectively. The AUCs of combined IFN-β and PLT were 0.81 at acute phase and 0.96 at subacute phase of KD, with sensitivity and specificity of 97.22 and 55.26%, and 86.36 and 100%, respectively. The cutoff value of combined IFN-β and PLT detection was IFN-β = 3.51 pg/ml and PLT = 303 × 10^9^/L at acute phase of KD, IFN-β = 4.21 pg/ml and PLT = 368 × 10^9^/L at subacute phase from plot vs. criterion values. However, there are no significant differences between the CAD– group and the CAD+ group for combined IFN-β and PLT, both *P* > 0.5, neither at acute nor at subacute phase of KD.

**Conclusion:** Combined IFN-β and PLT detection is an efficient biomarker for KD identification. The cutoff values are IFN-β = 3.51 pg/ml and PLT = 303 × 10^9^/L at acute phase of KD and IFN-β = 4.21 pg/ml and PLT = 368 × 10^9^/L at subacute phase.

## Introduction

Kawasaki disease (KD) is an acute febrile disease, mainly manifested as systemic vasculitis. It is a self-limiting disease, with its etiology still unknown. The main complication of KD is cardiovascular complications, such as potential occlusion and cardiac ischemia caused by coronary artery aneurysm (CAA). KD is the primary cause of acquired heart disease in children. However, how to diagnose and recognize KD in the early stage is very important for clinicians, especially those atypical KD. Researchers showed that delayed diagnosis of KD was an elevated risk of CAA (1).

The cause of KD was still not very clear. Recent evidence suggests interplay between a microbial infection and a genetic predisposition in the development of the disease. Giordani et al. (2) confirmed the hypothesis of an infectious triggered overexpression of inflammatory cytokines in KD. Many known and unknown infectious agents have been linked to KD, such as bacterial, viral, and fungal agents, and whether a single as-yet unidentified agent is responsible or whether multiple agents can produce a similar pattern of symptoms in genetically predisposed individuals was unknown (3).

The diagnosis of KD mainly depended on clinical manifestation. According to the 2017 American Heart Association (AHA) management for KD, the typical KD manifestations should include skin rash, bilateral conjunctival hyperemia, chapped lips, strawberry tongue, neck lymphadenopathy, and peeling of the fingers and toes (4). Typical KD could be recognized during the early period, whereas, the atypical KD was often missed. So, it is important to find the early biomarker of KD. The identification of inflammatory, proteomic, and genetic biomarkers may assist in earlier and more effective diagnosis and treatment. Parthasarathy et al. (5) suggested that N-terminal pro–B-type natriuretic peptide was a very promising biomarker for future investigation. Usually, platelets (PLTs) may increase specifically during subacute and convalescent phases of KD (6). It is commonly thought to play a role in hemostasis and thrombosis (7) and may be contributed to intravenous immunoglobulin (IVIG) resistance and CADs (8, 9). Zheng et al. (10) found that lower platelet distribution Width (PDW) and mean platelet volume (MPV) may be available markers for early diagnosis of KD.

Interferons (IFNs), discovered in the 1950s, represent a family of cytokines, which induce robust antiviral and immunomodulatory responses to interfere with virus replication and spread. IFN-α and IFN-β are type I IFNs; all nucleated cells, especially plasmacytoid dendritic cells, can produce IFN-α and IFN-β when virus is infected (11). Several studies revealed that type I IFN signaling is involved in the production of proinflammatory cytokines and the activation of inflammasome during certain bacterial and viral infections (12–15). Rowley et al. (16) confirmed that the immune transcriptional profile in KD coronary artery tissues has features of an antiviral immune response such as activated cytotoxic T lymphocyte and type I IFN–induced gene upregulation through RNA sequencing of KD coronary arteries. In our study, we try to evaluate the value of combined detection of IFN-β and PLT for KD.

## Methods

We have used three cohorts: KD cohort and two independent control cohorts including febrile control and normal healthy control.

### KD Patients

Forty-four patients diagnosed with KD for the first time upon admission to the children's hospital affiliated with Soochow University between April 2019 and December 31, 2019, were enrolled in this study as KD cohort (A) (26 male and 18 female patients). Patients were enrolled if they met the 2017 and 2019 AHA diagnosis guidelines for KD and atypical KD. All KD group children took echocardiography examination twice both at the second day of admission and 3 days after the body temperature became normal. According to the management of the 2017 AHA guidelines (4), we divided the KD group into without coronary artery disease group (CAD–) and with coronary artery disease group (CAD+). We had 30 cases of CAD– and 14 cases of CAD+, including three cases of mild coronary artery dilation, nine cases of medium aneurysm, and two cases of large aneurysm. Our study obtained the approval of the ethics committee of Soochow University Affiliated Children's Hospital (no. 2020CS087).

Peripheral blood samples of KD group were collected at acute phase before IVIG (3–5 days of fever) and at subacute phase of KD after IVIG (3 days after fever subsided).

### Febrile Patients

Forty-four children admitted to our hospital suffering from other febrile illnesses were selected as a febrile control cohort (B) hospitalized at the same period with the KD cohort (28 male and 16 female patients). Thirty-seven of them were admitted for pneumonia, one for bronchitis, five for acute tonsillitis, and one for upper respiratory tract infection. Peripheral blood samples of febrile group were collected at 3–5 days of fever.

### Normal Healthy Control

After informed consent was obtained, 44 healthy children undergoing physical examinations were also selected as healthy control cohort (C) (21 male and 23 female patients) at the same period with the KD group and febrile group sample obtained.

### Analysis of IFN-β and PLT

IFN-β was measured by high-throughput liquid-phase protein chip detection at Laizi Biotechnology Co., Ltd. (cat. no. PPX-08-MXNKTAU, lot no. 230611-00). The interassay coefficient of variation (CV) and intra-assay CV of the kit are both >15%. The plate was washed with 150 μL 1× wash buffer in the kit each time. (1) Reagent and standard dilution is operated according to the protocol provided in the kit. (2) Microspheres were prepared: 50 μl of premixed microspheres was added to each 96-well plate. The 96-well plate was placed into the magnetic separation was let to stand still for 2 min until microspheres sink to the bottom. Then, the magnetic plate was quickly turned upside down, and the liquid was poured out in the hole. The 96-well plate was not removed from the magnetic separation during this process; 150 μl 1× wash buffer was added to each well and let stand for 30 s, and then the magnetic plate was turned upside down to remove the liquid. A paper towel was used to absorb liquid that remained on the surface. (3) Microspheres and sample incubation: 25 μl universal assay buffer and sample were added to each well; 25 μl universal assay buffer was added to the blank control; orifice sealing film, 500 rpm for 30 min at room temperature, and was let to stand overnight at 4°C. This was taken out the next day and incubated with shaking at 500 rpm for 30 min at room temperature. (4) Plate was washed. (5) Detection antibody was added: 25 μl 1× detection antibody mixture was added to each well; a new sealed well-plate was used; the 96-well plate was removed from the magnetic separation and was shaken in the well-plate shaker at 500 rpm for 30 min at room temperature. (6) Plate was washed. (7) SA-PE was added: 50 μl SA-PE was added to each well; a new sealed membrane orifice plate was used; the 96-well plate was removed from the magnetic separation. As for the orifice plate shaker, it was shaken at 500 rpm for 30 min at room temperature. (8) Plate was washed. (9) On-board testing: 120 μl reading buffer was added to each well; well-plate with a new membrane was sealed; the 96-well plate was removed from the magnetic separation and was shaken in a shaker at 500 rpm at room temperature for 5 min; the sealing membrane was gently removed and put it into a Luminex 200 instrument for reading. Result was analyzed by a five-parameter non-linear regression method and expressed in pg/ml.

PLT was measured *via* flow cytometry (Sysmex and Mindray, matching kit).

### Statistical Analysis

For comparison between count data, when the expected frequency of each grid is *E* ≥ 5 and *n* ≥ 40, the Pearson χ^2^ test is used; when the comparison is *E* < 5 and/or *n* < 40, the Fisher exact test is used. *P* < 0.5 means the difference is statistically different. Sample dates were not normally distributed; the Mann–Whitney *U*-test was used for the mean of two related samples; the Kruskal–Wallis test was used for multiple sample comparisons. The comparison between groups was performed by the Conover method for pairwise comparison. The results were described by the median or 95% confidence interval (CI), and *P* < 0.05 had a statistically significant difference.

A logistic regression analysis with stepwise selection was used to examine the predictive value [odds ratio (OR)] of the IFN-β and PLT for acute KD vs. fever and health control groups and for subacute KD vs. fever and health control groups. Receiver operating characteristic (ROC) curves were plotted from the predictive markers derived from the logistic regression analysis. The area under the ROC curve (AUC) from both the individual candidate markers and combination of candidate markers (the predicted probability) was used to check for discriminatory value. The best discriminatory cutoff value was calculated with plot vs. criterion values. The cutoff from the discovery cohort was applied to the validation cohorts, and the corresponding sensitivity and specificity with 95% CI were calculated. Using predicted probabilities based on the discovery model, ROC curves in the discovery model were plotted, and AUCs were compared to the AUCs from the discovery model. MedCalc 15.2.2 was used for all statistical analyses.

## Results

### Manifestation

The age at onset in the KD cohort was significantly lower than that of the febrile control cohort, and the difference between the groups was statistically significant, *P* = 0.03, *P* < 0.5. The gender and number of fever days in the KD group had no significant difference between the febrile control group and the healthy control group, *P* > 0.05. There was no significant difference in the age at onset, days of fever, and gender between CAD– and CAD+ group, *P* > 0.05 (Table 1).

**Table 1:** Demographic data of Kawasaki disease (KD) patients and two control groups.

	**KD (*****n*** **=** **44)**				**Febrile control (*n* = 44)**	**Healthy control (*n* = 44)**	**Z/χ^2^**	***P***
	**CAL–(*n* = 30)**	**CAL + (*n* = 14)**	**Z/χ^2^**	***P***	**-**	**-**	**-**	
IVIG non-responders/responders	2/28	0/14	-	-	-	-	-	
Complete/incomplete KD	29/1	14/0	-	-	-	-	-	
Pneumonia	-	-	-	-	37	-	-	
Bronchitis	-	-	-	-	1	-	-	
Acute tonsillitis	-	-	-	-	5	-	-	
Upper respiratory infection	-	-	-	-	1	-	-	
Age at onset (m, 95% CI)	23.87–49.00	13.79–41.10	1.42	0.15	35.03–50.97	36–47.58	2.24	0.03
Duration of fever (d, 95% CI)	5.00–6.00	4.00–6.10	0.49	0.63	5.00–6.00	/	0.56	0.58
Male/female^a^	18/12	8/6	0.02	0.88	28/16	21/23	2.40	0.29

a *Person Chi-square test*.

The manifestations of KD included rash (75%), bilateral conjunctival congestion (86.36%), lip congestion or chapping (86.36%), strawberry tongue (81.82%), cervical lymphadenopathy (88.64%), swelling of the hands and feet (52.27%), BCG scar hyperemia (11.36%), and finger/toe peeling (27.27%). There was no significant difference in the clinical manifestations between the CAD– and the CAD+ group, all *P* > 0.5 (Table 2).

**Table 2:** Clinical characteristics of patients of Kawasaki disease (KD) patients.

	**CAL-(*n* = 30)**	**CAL + (*n* = 14)**	***P***
Rash	22/8	11/3	1
Conjunctivitis	26/4	12/2	1
Oral changes	26/4	12/2	1
Strawberry tongue	23/7	13/1	0.40
Cervical lymphadenopathy	28/2	11/3	0.31
Extremity changes^a^	17/13	6/8	0.07
BCG scar	5/25	0/14	0.16
Peeling	9/21	3/11	0.72

a *Person Chi-square test, χ^2^ = 0.281*.

### Comparisons of IFN-β and PLT Between KD Cohort vs. Febrile Control and Healthy Control Cohorts

In this study, we try to test the values of IFN-β and PLT in predicting KD against febrile disease. We found that both IFN-β and PLT at subacute phase of KD were higher than those at acute phase; the difference between them had statistical significance, *P* < 0.5 (Table 3). The concentrations of IFN-β and PLT are higher than those of the febrile control group and healthy control group both at acute and subacute phases of KD, and their difference had statistical significance, both *P* < 0.05 (Tables 4, 5).

**Table 3:** Comparison of IFN-β and PLT between acute phase of KD and subacute phase of KD.

	**Acute phase of KD (A, 95% CI)**	**Subacute phase of KD (B, 95% CI)**	**Z**	***P***
IFN-β (pg/ml)	3.32–4.69	4.50–5.72	−2.72	0.01
PLT (*10^∧^9/l)	316.28–381.97	484.55–574.90	−5.59	<0.00

**Table 4:** Comparison of IFN-β and PLT in the acute phase of KD (A), febrile control group (C), and healthy control group (D).

	**The acute phase of KD (A, M)**	**Febrile control group (C, M)**	**Healthy control group (D, M)**	***P***	**A/C**	**A/D**	**C/D**
IFN-β (pg/ml)	3.93	2.565	1.9	0.00	Yes	Yes	No
PLT (*10^∧^9/l)	359	270.5	306	0.00	Yes	Yes	No

**Table 5:** Comparison of IFN-β and PLT in the subacute phase of KD (B), febrile control group (C), and healthy control group (D).

	**The subacute phase of KD (B, M)**	**Febrile control group (C, M)**	**Healthy control group (D, M)**	***P***	**B/C**	**B/D**
IFN-β (pg/ml)	31.25	2.565	1.9	<0.00	Yes	Yes
PLT (*10^∧^9/l)	544.5	270.5	306	<0.00	Yes	Yes

### Logistic Regression Analysis

Logistic regression analysis was used to evaluate the value of combined IFN-β and PLT for KD. During acute phase of KD, the overall model fit by Hosmer and Lemeshow test was well, χ^2^ = 16.68, *P* > 0.5. Both IFN-β and PLT could be used to diagnose KD, *P* < 0.5 by Wald test, and OR = 1.43, 95% CI = 1.08–1.89 for IFN-β, and OR = 1.01, 95% CI = 1.00–1.02 for PLT.

At subacute phase of KD, the overall model fit was also well by Hosmer and Lemeshow test, χ^2^ = 8.98, *P* > 0.5. Both IFN-β and PLT contributed to KD, *P* < 0.5 by Wald test, and OR = 1.47, with 95% CI = 1.12–1.94 for IFN-β, OR = 1.03, with 95% CI = 1.01–1.04 for PLT (Tables 6, 7).

**Table 6:** Logistic regression analysis and AUC of IFN-β and PLT for acute and subacute phase of Kawasaki disease.

	**β**	***P***	**OR**	**95% CI**	**AUC**	**Sensitivity%**	**Specificity%**
IFN-β-A	0.36	0.01	1.43	1.08–1.89	0.81	97.22	57.89
PLT-A	0.01	0.01	1.01	1.00–1.02	0.71	63.64	73.86
IFN-β-B	0.39	0.01	1.47	1.12–1.94	0.88	97.73	68.42
PLT-B	0.03	0.00	1.03	1.01–1.04	0.93	81.82	95.45

*IFN-β-A, IFN-β at acute phase of KD; IFN-β-B, IFN-β at subacute phase of KD; PLT-A, PLT at acute phase of KD; PLT-B, PLT at subacute phase of KD; β, regression coefficient*.

**Table 7:** Logistic regression analysis and AUC for IFN-β and PLT combined detection at acute and subacute phase of Kawasaki disease.

	**Hosmer & Lemeshow test**		**AUC**	**Youden index**	**Sensitivity (%)**	**Specificity (%)**	***p***
	**χ^2^**	***P***					
IFN-β-A + PLT-A	16.68	0.05	0.81	0.52	97.22	55.26	<0.0001
IFN-β-B + PLT-B	8.98	0.34	0.96	0.86	86.36	100	<0.0001

### ROC Analysis

ROC curves were used for the predictive ability of IFN-β, PLT, and combined IFN-β and PLT to discriminate between acute phase of KD, subacute phase of KD, and febrile disease.

**Figure 1:**
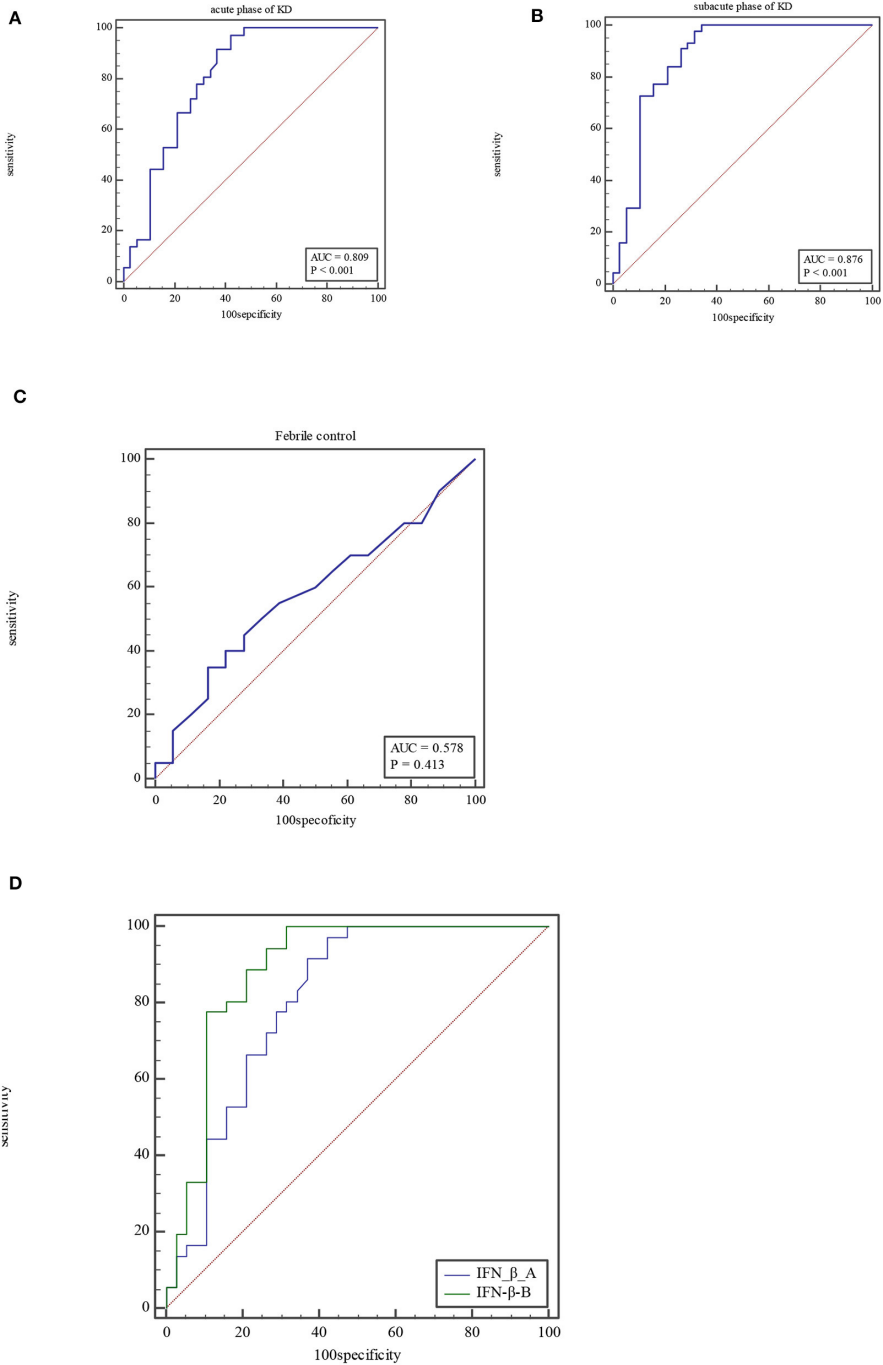
ROC curve of IFN-β at acute phase of KD **(A)**, subacute phase of KD **(B)**, and febrile control corhort **(C)**. Comparison of ROC for IFN-β between at acute phase of KD and subacute phase **(D)**.

For IFN-β, the AUC was 0.81 (95% CI = 0.70–0.89, *P* < 0.0001), with a sensitivity of 97.22% and specificity of 57.89% and cutoff of >2.5 pg/ml at acute phase of KD (Figure 1A). At subacute phase of KD, the AUC was 0.88 (95% CI = 0.79–0.94, *P* < 0.0001), with a sensitivity of 97.73% and specificity of 68.42% and cutoff of >2.9 pg/ml (Figure 1B). In the febrile control group, the AUC was 0.58 (95% CI = 0.407–0.74, *P* = 0.41), with a sensitivity of 35% and specificity of 83.33% and cutoff of >3.15 pg/ml (Figure 1C). Comparison of ROC curves between acute phase and subacute phase of KD revealed that the difference of AUC is about 0.08, *Z* = 2.96, *P* = 0.0031 (Figure 1D).

**Figure 2:**
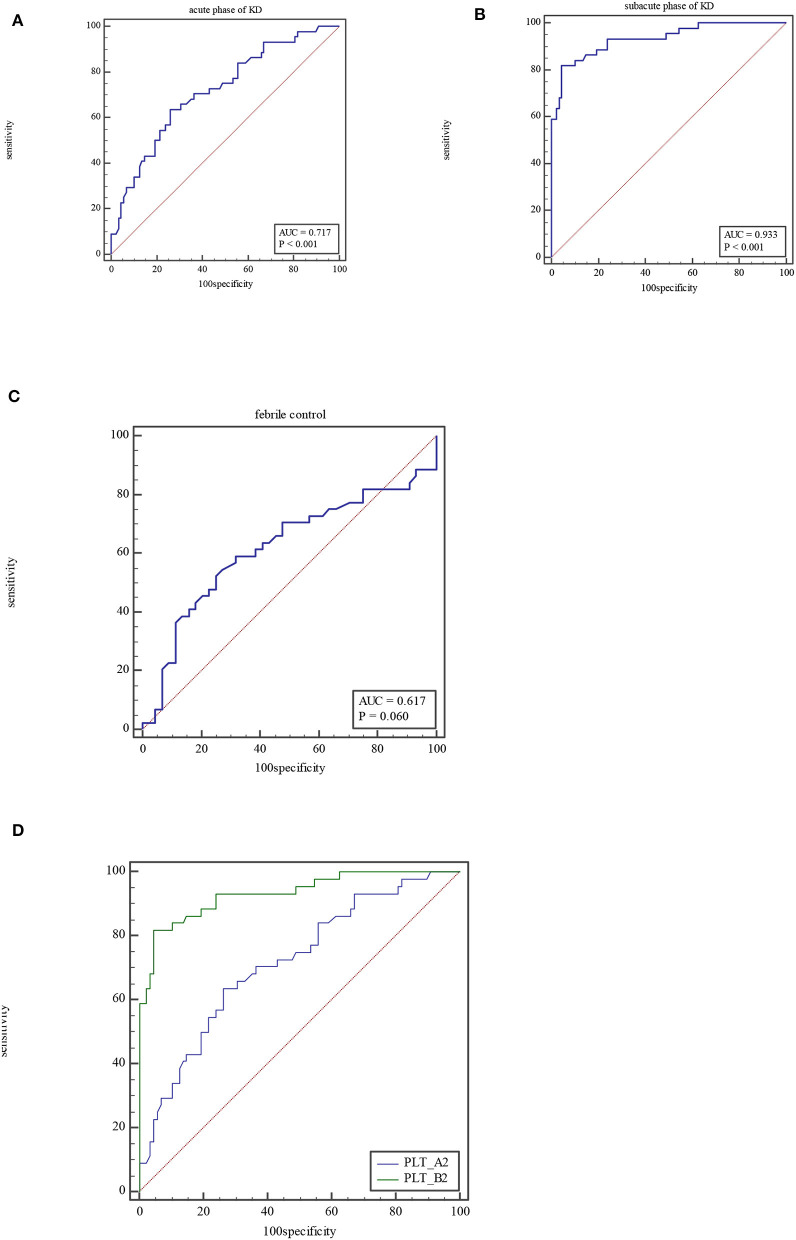
ROC curve of PLT at acute phase of KD **(A)**, subacute phase of KD **(B)**, and febrile control corhort **(C)**. Comparison of ROC for PLT between at acute phase of KD and subacute phase **(D)**.

For PLT, the AUC was 0.72 (95% CI = 0.63–0.79, *P* < 0.0001), with a sensitivity of 63.64% and specificity of 73.86% and cutoff of >322 × 10^9^/L at acute phase of KD (Figure 2A). At subacute phase of KD, the AUC was 0.93 (95% CI = 0.88–0.97, *P* < 0.0001), with a sensitivity of 81.82% and specificity of 95.45% and cutoff of >423 × 10^9^/L (Figure 2B). In the febrile control group, the AUC was 0.62 (95% CI = 0.51–0.72, *P* = 0.06), with a sensitivity of 52.27% and specificity of 75% and cutoff of ≤272 × 10^9^/L (Figure 2C). Comparison of ROC curves between acute phase and subacute phase of KD revealed that the difference of AUC is about 0.21, *Z* = 0.556, *P* < 0.0001 (Figure 2D).

**Figure 3:**
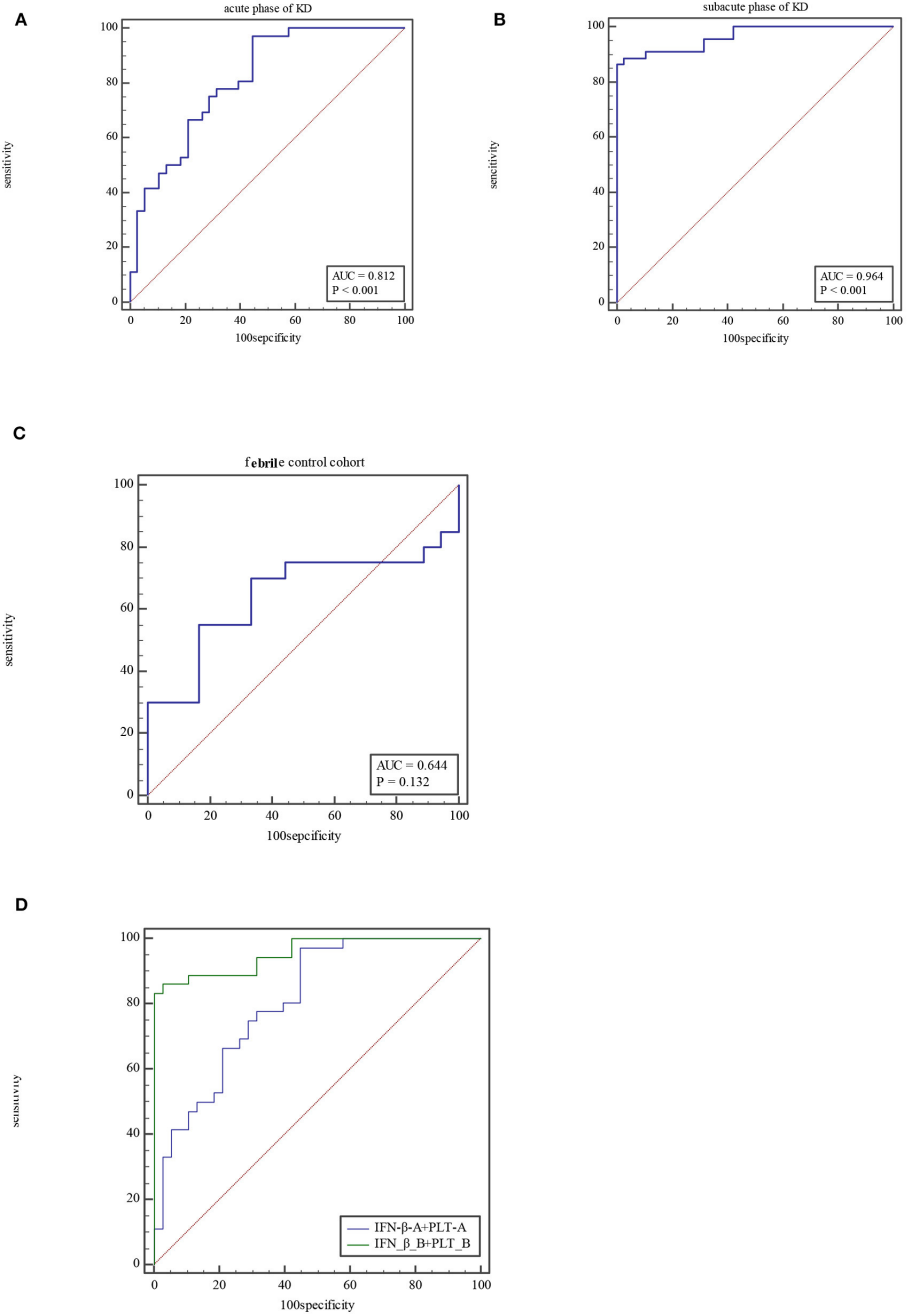
ROC for combined IFN-β and PLT detection at acute phase of KD **(A)**, subacute phase of KD **(B)**, and febrile control corhort **(C)**. Comparison of ROC for combined IFN-β and PLT detection between at acute phase of KD and subacute phase **(D)**.

**Figure 4:**
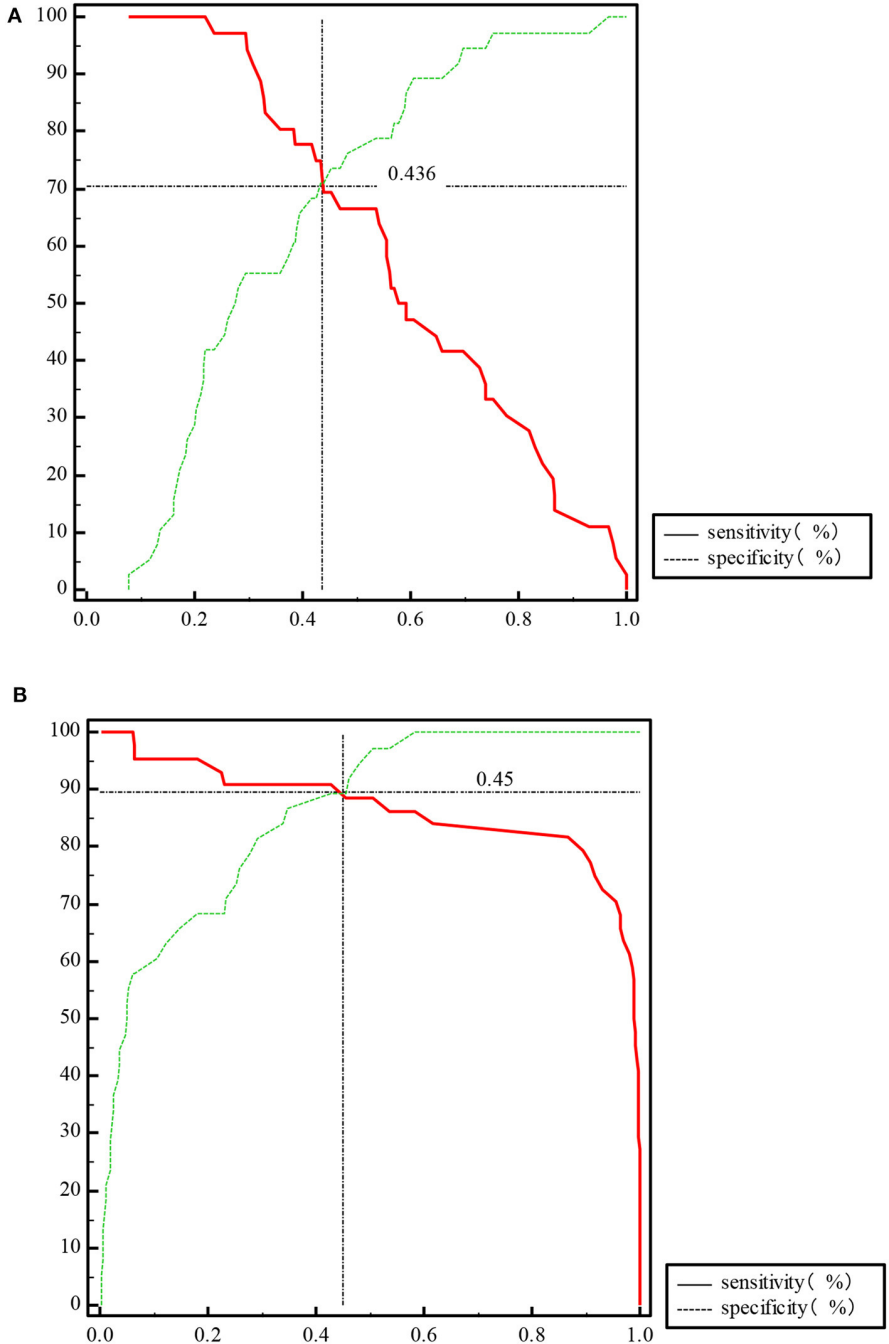
Plot vs. criterion values of propensity for IFN-β and PLT combined detection at acute phase of KD **(A)** and subacute phase of KD **(B)**.

The ROC curve of the predicted probability of KD with IFN-β and PLT combined detection was 0.81 (95% CI = 0.70–0.89, *P* < 0.0001), with a sensitivity of 97.22% and specificity of 55.26% (Figure 3A). At subacute phase of KD, the AUC was 0.96 (95% CI = 0.897–0.992, *P* < 0.0001), with a sensitivity of 86.36% and specificity of 100% (Figure 3B). In the febrile control group, the AUC was 0.64 (95% CI = 0.47–0.79, *P* = 0.13), with a sensitivity of 55% and specificity of 83.33% (Figure 3C). Comparison of ROC curves between acute phase and subacute phase of KD revealed that the difference of AUC is about 0.14, *Z* = 3.68, *P* = 0.0002 (Figure 3D). Plot vs. criterion values for combined IFN-β and PLT detection were about 0.44 at acute phase and 0.45 at subacute phase. The cutoff values of IFN-β and PLT were IFN-β = 3.51 pg/ml and PLT = 303 × 10^9^/L at acute phase of KD, and IFN-β = 4.21 pg/ml and PLT = 368 × 10^9^/L at subacute phase. When IFN-β ≥3.51 pg/ml and PLT ≥303 × 10^9^/L, the specificity for KD would rise with decreased sensitivity. Otherwise, when IFN-β <3.51 pg/ml and PLT <303 × 10^9^/L, the specificity would decrease, but the sensitivity would rise (Figure 4).

### Comparisons of IFN-β, PLT, and IFN-β and PLT Combined Detection Between the CAD– and CAD+ Groups

There was no statistical difference between CAD– and CAD+ group for IFN-β, PLT, and IFN-β and PLT combined detection both at acute and subacute phases of KD, *P* > 0.5.

## Discussion

### KD and Manifestation

KD is an acute, self-limited febrile disease that predominantly affects children <5 years of age. The diagnosis of typical KD is based on the presence of ≥5 days of fever (first calendar day of fever is illness day 1) and the presence of ≥4 of the five principal clinical features, including (1) erythema and cracking of lips, strawberry tongue, and/or erythema of oral and pharyngeal mucosa; (2) bilateral bulbar conjunctival injection without exudate; (3) rash: maculopapular, diffuse erythroderma, or erythema multiforme–like; (4) erythema and edema of the hands and feet in acute phase and/or periungual desquamation in subacute phase; (5) cervical lymphadenopathy (≥1.5-cm diameter), usually unilateral. In the presence of more than four principal clinical criteria, particularly when redness and swelling of the hands and feet are present, the diagnosis may be made with only 4 days of fever (4). Patients who meet the principal clinical findings are said to have complete KD (typical or classic KD). Patients who do not have sufficient principal clinical findings may be diagnosed with incomplete KD (atypical KD). The clinical findings may not appear at the same time; their appearance was sometimes individualized and hysteretic. In our study, we had rash (75%), bilateral bulbar conjunctival congestion (86.36%), lip congestion, or chapping (86.36%), strawberry tongue (81.82%), neck lymphadenopathy (88.64%), swelling of the hands and feet (52.27%), BCG scar hyperemia (11.36%), and peeling of fingers and toes (27.27%). BCG scar hyperemia is not currently recognized as a primary factor for KD diagnosis, but it can be used as an early diagnostic symptom for KD. Garrido-García et al. (17) suggest that BCG site reactivation can serve as a valuable indicator for KD diagnosis. Rezai et al. (18) found that BCG site reactivation was more commonly observed in children with KD at younger than 2 years than lymphadenopathy. We also observed 80% of BCG scar hyperemia incidence among children younger than 2 years. Therefore, we support that BCG site reactivation can be used for early diagnosis of KD.

The three cohorts of children in this study were all younger than 5 years, and the median values of the three groups were 33, 43, and 36 months, respectively. The age at onset of the KD group was significantly lower than that of the fever control group. However, the gender and number of fever days in the KD group had no significant difference from the febrile control group and the healthy control group. This indicates that KD may emerge in lower-age children. However, this difference also can be caused by selection bias.

### PLT and KD

PLT is a useful biomarker for KD. It is usually increased at subacute phase of KD. We also found that patients with KD have much higher level of PLT than the febrile control group. Comparison of ROC curves revealed that PLT is more sensitive and specific at subacute phase than at acute phase. We recommend PLT >322 × 10^9^/L at acute phase and >423 × 10^9^/L at subacute phase for KD prediction. However, a retrospective, cross-sectional study showed that aspartate aminotransferase, alanine aminotransferase, and PLT had a significant but low sensitivity for KD diagnosis (19). A multicenter study revealed that lymph node enlargement, erythrocyte sedimentation rate ≥75 mm/h, and PLT ≥530 × 109 /L are independent risk factors for predicting non-response to sufficient IVIG doses (20). Zhou et al. (21) concluded that vascular endothelial growth factor, interleukin 6 (IL-6), PLT, and d-dimer were the important risk factors for KD complicated with CAD.

### IFN-β and KD

IFN-β is a member of type I IFNs, secreted by all nucleated cells after viral infection. The DNA or RNA of the pathogen can be recognized by the pattern recognition receptors on the cell surface and activated cyclic guanosine monophosphate–adenosine monophosphate synthase (cGAS) through different pathways, which can catalyze ATP and GTP into cyclic monophosphate–adenosine monophosphate (cGAMP). cGAS can induce the production of IFN-β by a STING-dependent manner. STING is located on the endoplasmic reticulum membrane, and the outer mitochondrial membrane contains 4-transmembrane structure of N-terminal domain and C-terminal domain (CTD). The combination of C-terminal tail (CTT) and CTD keeps STING in inhibited state at the resting condition. cGAMP as a second messenger can bind to the dimerized STING and separate the CTT-CTD of STING, which in turn activates STING. The activated STING migrates from the endoplasmic reticulum to the punctate membrane structure around the nucleus through the Golgi apparatus and then recruit TANK-binding kinase 1 (TBK1) and IFN regulator factor 3 (IRF3). IRF3 phosphorylated by TBK1 enters into the nucleus, inducing the production of IFN-α, IFN-β, IFN-γ, IL-2, IL-12, and other cytokines, and promotes the proliferation and differentiation of T and B cells (22).

**Figure 5:**
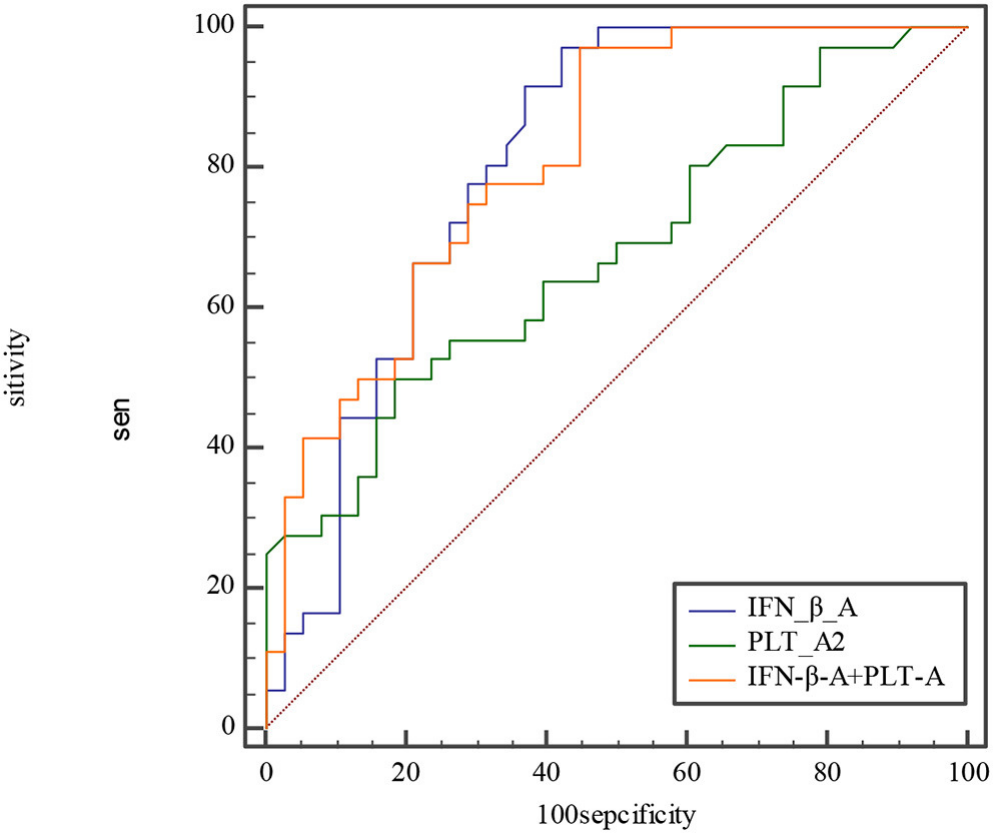
Comparison of ROC at acute phase between IFN-β, PLT, and combined IFN-β and PLT. The AUC of IFN-β, PLT, and combined IFN-β and PLT is 0.809, 0.679, and 0.812, with 0.701–0.891, 0.560–0.783, 0.704–0.894 for their 95% CI separately. The difference of AUC between IFN-β and PLT is about 0.13, Z≈1.57, *P*≈0.12. The difference of AUC between IFN-β and combined IFN-β and PLT is about 0.003, Z≈0.08, *P*≈0.93. The difference of AUC between PLT and combined IFN-β and PLT is about 0.13, Z≈2.558, *P*≈0.01.

**Figure 6:**
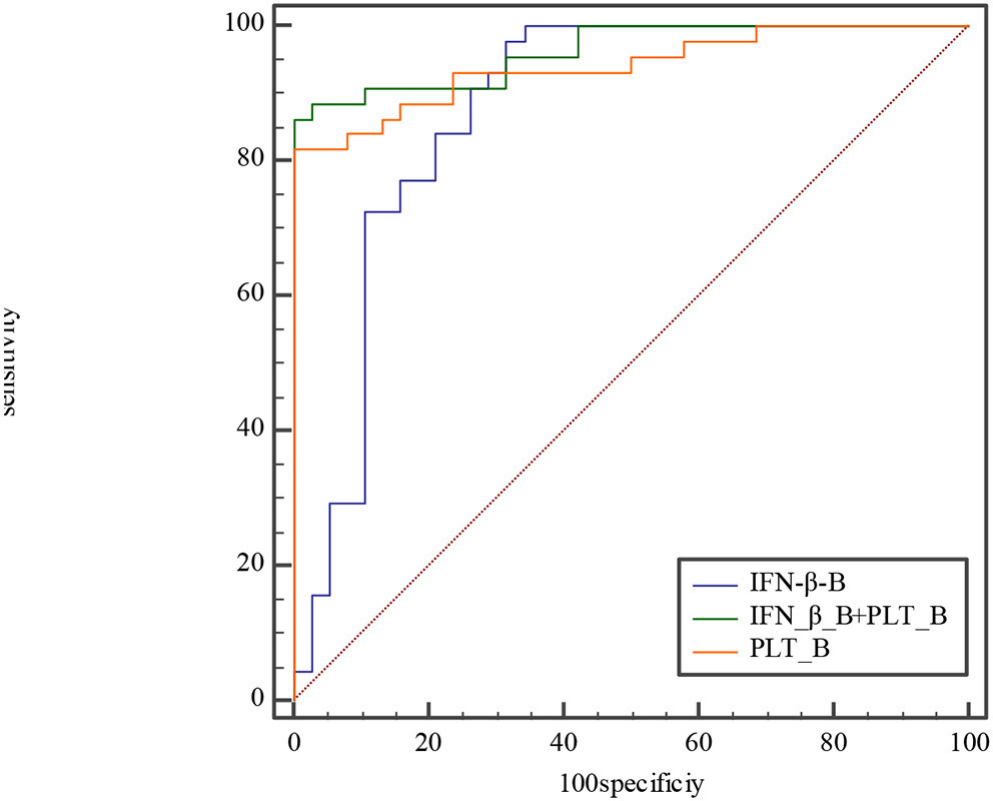
Comparison of ROC curve at subacute phase between IFN-β, PLT, and combined IFN-β and PLT. The AUC of IFN-β, PLT, and combined IFN-β and PLT is 0.876, 0.941, and 0.964, with 0.785–0.939, 0.866–0.981, 0.897–0.992 for their 95% CI separately. The difference of AUC between IFN-β and PLT is about 0.06, Z≈1.28, *P*≈0.20. The difference of AUC between IFN-β and combined IFN-β and PLT is about 0.09, Z≈2.1, *P*≈0.04. The difference of AUC between PLT and combined IFN-β and PLT is about 0.02, Z≈1.89, *P*≈0.06.

The etiology and pathogenesis of KD are still unknown. Some studies have confirmed that the epidemiology of KD and the higher incidence of CAD are both related to infection (23–25). Superantigens produced by bacteria (26, 27), viruses (28–30), atypical pathogens (31), and fungi (32) may trigger immune disorder and cause KD. In the 2019 coronavirus disease (COVID-19) pandemic, a large number of children with KD-like symptoms appeared in the United Kingdom, the United States, and Italy. A systematic review showed that the COVID-19 epidemic was associated with an increased incidence of severe KD (33). Berthelot et al. (34) made a new hypothesis that the STING pathway is overactivated in COVID-19. We observed that both IFN-β and PLT at acute and subacute phases of KD are higher than those of the febrile control group and the healthy control group. Moreover, both at subacute phase of KD were higher than they were at acute phase, with higher sensitivity and specificity. According to comparison of ROC curves at acute phase between IFN-β, PLT, and combined IFN-β and PLT, we find that both IFN-β and IFN-β and PLT combined detection are more efficient for KD prediction than PLT (Figure 5). But at subacute phase of KD, combined IFN-β, and PLT detection is the most efficient for KD (Figure 6). The sensitivity and specificity of combined IFN-β and PLT detection is 97.22 and 55.26% at acute phase, and 86.36 and 100% at subacute phase, respectively. Based on this, we recommend that combined IFN-β and PLT is more efficient for KD identification. The cutoff values are IFN-β = 3.51 pg/ml and PLT = 303 × 10^9^/L at acute phase of KD and IFN-β = 4.21 pg/ml and PLT = 368 × 10^9^/L at subacute phase.

CAD is the most serious complication of KD. We found the increased secretion of IFN-β during KD, especially at subacute phase. Although, we did not ascertain its difference between the CAD– and CAD+ groups, but like PLT, the high level of IFN-β may be a risk factor for CAD, as IFN-β released by monocytes–macrophages through the STING pathway could promote hypercoagulability (34).

### Limitations

There are several limitations to our study, which are as follows: (1) we must admit that there is a possibility of false positives in hypothesis testing; (2) we did not have enough CAD patients to identify the relationship between IFN-β and CAD; (3) the febrile control cohort in our study mainly suffers from respiratory tract infection. We may enroll other febrile diseases, such as sepsis, and divide them into more specific classification, such as viral or bacterial infection.

### Conclusion

Combined IFN-β and PLT detection is a more specific biomarker for KD identification. IFN-β is newly used for KD prediction, and its relationship with CAD needs further research.

## Data Availability Statement

The raw data supporting the conclusions of this article will be made available by the authors, without undue reservation.

## Ethics Statement

The studies involving human participants were reviewed and approved by the Ethics Committee of Soochow University Affiliated Children's Hospital (No. 2020CS087). Written informed consent to participate in this study was provided by the participants' legal guardian/next of kin. Written informed consent was obtained from the minor(s)' legal guardian/next of kin for the publication of any potentially identifiable images or data included in this article.

## Author Contributions

All authors listed have made a substantial, direct and intellectual contribution to the work, and approved it for publication.

## Conflict of Interest

The authors declare that the research was conducted in the absence of any commercial or financial relationships that could be construed as a potential conflict of interest.
